# CRISPR Screening in Hepatocellular Carcinoma: From Tumor Progression to Immune Evasion and Therapeutic Resistance

**DOI:** 10.3390/ijms27104241

**Published:** 2026-05-10

**Authors:** Shixin Ma, You Li, Teng Fei

**Affiliations:** 1 Interdisciplinary Research Center for Brain-Computer Interface, Key Laboratory of Bioresource Research and Development of Liaoning Province, College of Life and Health Sciences, Northeastern University, Shenyang 110819, China; mashixin0902@163.com (S.M.); nanjiliyou@163.com (Y.L.); 2 Foshan Graduate School of Innovation, Northeastern University, Foshan 528311, China; 3 National Frontiers Science Center for Industrial Intelligence and Systems Optimization, Northeastern University, Shenyang 110819, China; 4 Key Laboratory of Data Analytics and Optimization for Smart Industry, Northeastern University, Ministry of Education, Shenyang 110819, China

**Keywords:** liver cancer, hepatocellular carcinoma, CRISPR, tumor progression, immune evasion, drug resistance

## Abstract

Hepatocellular carcinoma (HCC) is the most common primary liver malignancy and a leading cause of cancer-related mortality worldwide. Despite advances in targeted therapies and immunotherapies, clinical outcomes remain poor owing to profound molecular heterogeneity, intrinsic therapeutic resistance, and complex immune evasion mechanisms. Although genomic profiling has identified recurrent alterations in HCC, large-scale functional validation of candidate drivers and vulnerabilities remains challenging. CRISPR (clustered regularly interspaced short palindromic repeats)-based screening technologies have transformed this landscape by enabling systematic interrogation of gene function in physiologically relevant contexts. In this review, we summarize recent studies that have applied CRISPR screening approaches in HCC research. These efforts have uncovered multilayered dependency programs that govern ferroptosis resistance, metabolic reprogramming, epigenetic regulation, tumor suppressor networks, immune evasion, and resistance to targeted therapies. We also discuss the major limitations of current studies, including model bias, incomplete representation of HCC heterogeneity, and technical constraints intrinsic to pooled screening. Overall, integration of CRISPR screening with patient-derived models, single-cell readouts, and precision editing technologies is expected to accelerate mechanistic discovery and biomarker-guided therapeutic prioritization for HCC.

## 1. Introduction

Hepatocellular carcinoma (HCC) represents the most prevalent primary liver malignancy and a leading cause of cancer-related mortality worldwide [1,2]. Its incidence continues to rise owing to diverse etiologies, including chronic hepatitis B virus (HBV) or hepatitis C virus (HCV) infection, alcohol-associated liver disease, aflatoxin exposure, and the growing burden of nonalcoholic fatty liver disease (NAFLD)-associated steatohepatitis [3]. Although surveillance, surgical resection, local ablation, transplantation, targeted therapies, and immunotherapies have improved management in selected patients, the prognosis of advanced HCC remains poor [1,4]. Clinical outcomes are constrained by late diagnosis, therapeutic resistance, and a complex tumor microenvironment that promotes immune escape [4,5,6].

Large-scale genomic and transcriptomic studies have identified recurrent alterations and greatly improved our understanding of HCC molecular classification [7,8,9]. However, genomic analyses alone cannot fully distinguish functional drivers from passenger events or define which genes are required for tumor maintenance under specific therapeutic and microenvironmental conditions. Therefore, scalable approaches that directly interrogate causal dependencies are needed.

Clustered regularly interspaced short palindromic repeats (CRISPR) and CRISPR-associated nuclease 9 (Cas9) technologies have transformed functional genomics by enabling systematic, genome-scale interrogation of gene function in physiologically relevant settings [10,11,12]. In HCC, pooled CRISPR knockout, CRISPR activation (CRISPRa), and CRISPR interference (CRISPRi) screens have enabled identification of causal gene dependencies governing tumor growth, metabolic adaptation, ferroptosis resistance, immune evasion, and therapeutic response [13,14,15,16,17,18]. These context-specific vulnerabilities are often difficult to infer from genomic data alone.

CRISPR screening studies in liver cancer have moved from functional validation of candidate cancer genes to the discovery of clinically relevant dependencies in HCC. In vivo multiplexed CRISPR/Cas9 screening models revealed context-specific tumor suppressors (e.g., *Pten*, *Cdkn2a*) and oncogenic drivers (e.g., *Myc*) that contribute to hepatocarcinogenesis [13,14]. More recently, CRISPR screens are increasingly used to define tyrosine kinase inhibitor (TKI) response. Genome-wide CRISPR/Cas9 screens in HCC cell lines have uncovered key resistance genes (e.g., *KEAP1*, *NF1*, *DUSP9*, and *DUSP4*) that mediate resistance to sorafenib, lenvatinib, and regorafenib, while pharmacological targeting of MAPK/ERK signaling with MEK inhibitors (e.g., trametinib or selumetinib) effectively restored drug sensitivity [19,20,21]. Similarly, kinome-focused CRISPR screens in HCC cell lines have revealed actionable kinase dependencies, including CDK7 and CDK12, whose pharmacological inhibition (THZ1 or THZ531), particularly in combination with sorafenib, exerts significant anti-tumor effects [22,23]. Notably, CRISPR screening has identified rational combination partners for targeted therapies, such as EGFR inhibition to enhance lenvatinib efficacy, with validation in preclinical models, including liver cancer cell line-derived xenograft models, immunocompetent mouse models, and patient-derived xenograft models, and emerging clinical evidence in advanced HCC (NCT04642547) [24]. Emerging studies are also beginning to integrate CRISPR perturbation with transcriptomics and metabolomics, which may further refine pathway-level mechanisms and biomarker-guided therapeutic stratification [25,26].

While foundational CRISPR screening approaches in liver cancer have been reviewed [27,28,29,30,31], emerging studies published from 2023 onward remain to be comprehensively integrated. In this review, we summarize the major CRISPR screening strategies applied in HCC-related studies (especially since 2023) and discuss how these approaches have uncovered functional dependencies linked to ferroptosis resistance, metabolic reprogramming, epigenetic regulation, tumor suppressor networks, immune evasion, and resistance to targeted therapies. The central question addressed here is how CRISPR-based functional screening has reshaped our understanding of actionable dependencies in HCC, particularly those linked to tumor progression, immune escape, and therapeutic resistance. Accordingly, this review is organized around screening-derived biological and therapeutic insights, while methodological platforms and experimental models are discussed as contextual factors that influence hit discovery, interpretation, and translational relevance.

## 2. CRISPR Screening Approaches in HCC Research

### 2.1. CRISPR Knockout Screens

Pooled CRISPR/Cas9 knockout screening is widely used to define genetic requirements for HCC growth, fitness, and response to therapy. In this framework, Cas9 endonuclease is directed by single-guide RNAs (sgRNAs) to specific genomic loci, where it introduces DNA double-strand breaks. Repair by the error-prone nonhomologous end-joining pathway frequently generates insertions or deletions (indels) that disrupt open reading frames and abolish gene function [10,32]. For screening experiments, large-scale sgRNA libraries are commonly introduced into HCC cell lines or patient-derived models by lentiviral infection at low multiplicity, so that most cells receive a single perturbation. After library integration, cells are expanded and subjected to defined selection conditions, such as routine culture, anticancer drug treatment, or tumor growth in mice. The relative abundance of each sgRNA is then measured by next-generation sequencing, allowing quantification of sgRNA depletion for fitness genes and enrichment of resistance-associated sgRNAs [10,11,12,33].

### 2.2. CRISPRa Screens

CRISPRa screening relies on nuclease-dead Cas9 (dCas9) linked to transcriptional activation modules to increase expression of endogenous genes at targeted loci [34,35,36]. In contrast to loss-of-function strategies, this platform is well suited for uncovering genes whose elevated expression restrains malignant growth or improves drug responsiveness. Within HCC studies, CRISPRa-based approaches can complement knockout screens by identifying protective programs that buffer oncogenic signaling, restore differentiation-associated pathways, or enhance sensitivity to anticancer therapies [37].

### 2.3. CRISPRi Screens

CRISPRi uses dCas9 coupled to transcriptional repression domains to reduce gene expression without introducing DNA double-strand breaks [34,35,38]. Because this method enables graded and potentially reversible suppression, it is particularly informative for studying genes that are difficult to assess by complete knockout due to severe fitness defects or early lethality.

### 2.4. Precision-Editing-Based CRISPR Screens

Precision-editing-based screens, including base editor and prime editor screens, provide an additional layer of functional interrogation beyond conventional CRISPR knockout, CRISPRi, and CRISPRa approaches. Base editors enable programmable single-nucleotide substitutions without inducing double-strand breaks, whereas prime editors allow more flexible introduction of base substitutions, small insertions, and deletions [39,40,41,42]. These approaches are particularly useful for assessing the functional consequences of cancer-associated point mutations, regulatory variants, splice-site alterations, and drug-resistance mutations at base-level resolution [43,44,45]. Although their application in HCC remains limited, precision-editing-based screens represent an important future direction for modeling clinically relevant mutations and identifying mutation-specific vulnerabilities in HCC.

**Figure 1:**
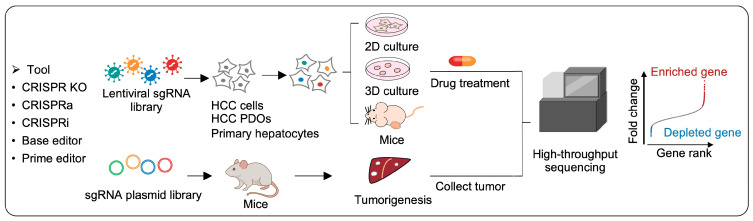
Overview of the CRISPR-based functional screening workflow in HCC. CRISPR libraries (knockout, activation, interference, base editor, or prime editor) are delivered into HCC cells, PDOs, or primary hepatocytes cultured in 2D/3D or mouse transplantation models. Cells are subjected to various selection pressures, including drug treatments (ferroptosis-related drugs, tyrosine kinase inhibitors). In vivo CRISPR screening can also be performed by direct injection of an sgRNA plasmid library into mouse livers to identify tumorigenesis-associated genes. Functional effects are assessed in vitro or in vivo followed by high-throughput sequencing of sgRNAs. Enriched genes (red) indicate perturbations that promote cell growth or confer resistance under the applied stress, whereas depleted genes (blue) represent genes whose loss impairs survival or sensitizes cells to specific stresses. CRISPR: clustered regularly interspaced short palindromic repeats; KO: knockout; CRISPRa: CRISPR activation; CRISPRi: CRISPR interference; sgRNAs: single-guide RNAs; HCC: hepatocellular carcinoma; PDOs: patient-derived organoids; 2D: two-dimensional; 3D: three-dimensional.

Collectively, these CRISPR screening strategies provide complementary loss-of-function, gain-of-function, and precision-editing modalities for functional interrogation in HCC-related studies (Figure 1).

### 2.5. Advanced Screening Strategies and Experimental Models

#### 2.5.1. FACS-Based CRISPR Screening

**Figure 2:**
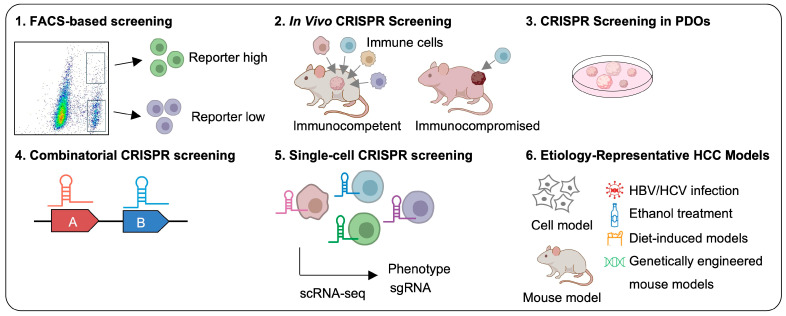
Schematic overview of advanced CRISPR-based screening strategies and experimental models. (1) FACS-based screening for enrichment of phenotype-associated cell populations; (2) in vivo CRISPR screening in immunocompetent and immunocompromised mouse models; (3) CRISPR screening in PDOs; (4) combinatorial CRISPR screening to investigate genetic interactions; (5) single-cell CRISPR screening to resolve cellular heterogeneity and perturbation-induced transcriptional states; (6) etiology-representative HCC models, including HBV/HCV infection, alcohol exposure, diet-induced models, and genetically engineered mouse models. FACS: fluorescence-activated cell sorting; scRNA-seq: single-cell RNA sequencing; HBV: hepatitis B virus; HCV: hepatitis C virus.

Several adapted CRISPR screening formats have expanded the range of phenotypes that can be interrogated (Figure 2). Screens combined with fluorescence-activated cell sorting (FACS) permit enrichment of cells according to reporter output, signaling activity, or cell-surface marker abundance [46]. CRISPRa screening has been applied to identify cellular transporter genes for special fluorescent probes that selectively distinguish malignant from healthy hepatocytes, providing a diagnostic tool for cancer detection in HCC [47].

#### 2.5.2. In Vivo CRISPR Screening

Screening results are highly dependent on biological context. Two-dimensional (2D) culture systems are experimentally convenient and have been effective for detecting cell-autonomous dependencies [33,48,49]; but they do not fully capture the structural and microenvironmental complexity of liver tumors. Accordingly, CRISPR screening in HCC has increasingly moved toward more physiologically relevant in vivo models. One commonly used strategy is to transduce human HCC cells with pooled sgRNA libraries ex vivo, followed by transplantation into mice for subcutaneous or orthotopic tumor screening, thereby enabling the identification of genes required for tumor growth in vivo. In parallel, in vivo CRISPR screens can also be performed by delivering sgRNA libraries directly through viral vectors or plasmid-based systems, allowing genetic perturbation in mouse livers within established tissue environments. Moreover, syngeneic models using murine HCC cells implanted into immunocompetent mice provide an important platform for interrogating tumor–immune interactions, immune surveillance, and immune evasion.

#### 2.5.3. PDO-Based CRISPR Screening Models

Patient-derived organoid (PDO)-based models are increasingly being used in HCC CRISPR screening. Although still less common than cell-line-based approaches, recent studies show that patient-derived HCC organoids can support both genome-wide and focused CRISPR knockout screens, allowing tumor dependencies to be interrogated in a three-dimensional (3D) setting that better preserves clinically relevant tumor features [50]. Organoids have also been adapted to model acquired drug resistance before screening, enabling the identification of context-specific and synthetic lethal vulnerabilities under therapeutic selection pressure [51].

#### 2.5.4. Multiplex Perturbation Screens for Genetic Interaction Mapping

CRISPR screening has evolved from single-gene perturbation to multiplex perturbation systems that permit systematic interrogation of genetic interactions. Barcoded pairwise sgRNA designs and Cas12a-based dual-CRISPR RNA (crRNA) architectures now make it possible to assess combinatorial perturbations at greater scale and with improved fidelity [25,52]. Such platforms are methodologically important because they move beyond single-gene dependency maps to identify synthetic lethal interactions, compensatory signaling relationships, and candidate target combinations that are more relevant to the redundancy of oncogenic networks.

#### 2.5.5. Single-Cell CRISPR Screening

Single-cell CRISPR screening links genetic perturbations to cell-state changes at high resolution. Perturb-seq and CROP-seq couple sgRNA identity with single-cell transcriptomic profiles, enabling systematic analysis of gene regulatory programs, cellular heterogeneity, and context-dependent dependencies [53,54]. Recent advances have further extended these approaches to multi-modal and spatial platforms, such as multi-omic Perturb-seq, ECCITE-seq, and spatial perturbation assays [55,56,57]. Although their application in HCC remains limited, these technologies offer a promising framework for dissecting tumor plasticity, immune regulation, and therapeutic vulnerabilities.

#### 2.5.6. Etiological Representation of HCC Models in CRISPR Screening Studies

Because HCC arises from diverse etiological backgrounds, including HBV infection, HCV infection, alcohol-associated liver disease, and NAFLD, the translational interpretation of CRISPR screening results depends on how faithfully these contexts are modeled. Although CRISPR/Cas9 screens have been performed in HBV- or HCV-related settings, most of identified genes are associated with viral entry, replication, or antigen production [58,59,60]. In contrast, HCV-, alcohol-, and NAFLD-associated HCC remain insufficiently represented in current screening platforms. Notably, a recent NAFLD-HCC-focused CRISPR/Cas9 screen identified *TUBB4B* as a potential selective dependency [61]. Future studies should incorporate more physiologically relevant systems, such as genetically engineered mouse models, or diet-induced NAFLD-HCC models, to better capture etiological heterogeneity.

## 3. Functional Landscapes of HCC Revealed by CRISPR Screening

**Figure 3:**
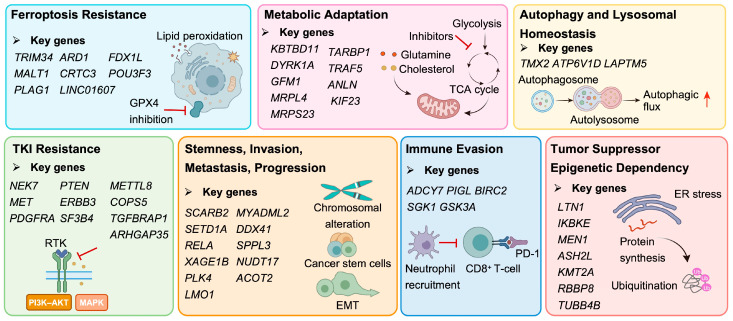
CRISPR screens reveal multilayered vulnerabilities and adaptive programs in HCC. Schematic overview of seven functional modules identified by CRISPR-based screening in HCC, including ferroptosis resistance, metabolic adaptation, autophagy and lysosomal homeostasis, therapeutic resistance, stemness/invasion/metastasis/progression, immune evasion, and tumor suppressor/epigenetic dependencies. Representative genes, pathways, and potential therapeutic vulnerabilities are highlighted. TKI: tyrosine kinase inhibitor; RTK: receptor tyrosine kinase; EMT: epithelial–mesenchymal transition; PD-1: programmed cell death protein-1.

To provide a structured overview of CRISPR-based functional screening in HCC, Figure 2 summarizes the principal screening strategies used to identify genetic regulators of tumor progression, cell-state adaptation, and therapeutic response, while representative studies, prioritizing those published from 2023 onward, are compiled in Table 1. The experimental systems summarized in Table 1 are heterogeneous in both biological origin and pathological classification. Although this review focuses on HCC, some cited CRISPR screening studies used nonconventional hepatic-origin models, mouse liver tumor models, primary hepatocytes, or auxiliary non-hepatic systems. These models were included because they were part of HCC-related screening workflows, and the key findings were subsequently validated in HCC-relevant cellular models. Collectively, these studies highlight several major functional themes, including ferroptosis resistance, metabolic reprogramming, epigenetic regulation, tumor suppressor networks, immune evasion, and resistance to targeted therapies. Rather than identifying isolated genes alone, these screens have begun to define the broader regulatory architecture underlying HCC progression and treatment failure (Figure 3).

### 3.1. Ferroptosis Resistance and Oxidative Stress Buffering: A Central Node in Therapeutic Vulnerability

Ferroptosis, an iron-dependent form of regulated cell death driven by lipid peroxidation, represents a promising therapeutic avenue for HCC. However, tumor cells can evade ferroptotic stress through antioxidant buffering, metabolic adaptation, and control of iron homeostasis. CRISPR-based screens have been instrumental in defining these resistance mechanisms and identifying actionable targets.

#### 3.1.1. GPX4-Centered Regulatory Networks in Ferroptosis Resistance

Genome-wide CRISPR screening has uncovered ferroptosis regulators with potential therapeutic relevance in HCC. In a Huh7 cell line-based 2D CRISPR knockout screen under erastin-induced ferroptotic stress, tripartite motif-containing protein 34 (*TRIM34*) emerged as a mediator of ferroptosis tolerance [62]. TRIM34 is upregulated in HCC tissues and associated with poor prognosis. Mechanistically, TRIM34 promotes the ubiquitination and degradation of Up-frameshift 1 (UPF1), thereby increasing GPX4 protein levels. *TRIM34* was further validated in nude mouse xenograft and experimental lung metastasis models, where its knockdown suppressed tumor growth and metastatic burden, whereas its overexpression had the opposite effects. Notably, *TRIM34* depletion sensitized HCC cells to anti-programmed cell death protein-1 (PD-1) therapy.

In parallel, an in vitro FACS-based whole-genome CRISPR screen identified *MALT1* as a determinant of GPX4 protein stability. MALT1 inhibition increased ring finger and CCCH-type domains 1 (RC3H1) expression, accelerated GPX4 ubiquitin-dependent degradation, and induced ferroptosis in liver cancer cells [63]. Notably, pharmacological targeting of MALT1 also cooperated with sorafenib to enhance ferroptosis in PDO models. Similarly, pleiomorphic adenoma gene 1 (*PLAG1*) was identified through CRISPR-based screening in HCC cells as a suppressor of sorafenib-induced ferroptosis, acting through GPX4-associated redox maintenance [64]. Beyond in vitro validation, *PLAG1* knockdown enhanced sorafenib efficacy in both subcutaneous and orthotopic xenograft models. Together, these studies highlight GPX4-centered regulatory networks as actionable vulnerabilities in HCC.

#### 3.1.2. Glutathione and Lipid Metabolic Control of Ferroptosis Sensitivity

Glutathione (GSH) serves as the essential cofactor for GPX4-mediated lipid peroxide detoxification, making its biosynthesis a critical determinant of ferroptosis sensitivity. Liu et al. integrated a whole-genome CRISPR/Cas9 screen in Huh7 cells with targeted metabolomics and identified acetyltransferase *ARD1* (N-alpha-acetyltransferase 10, *NAA10*) as a key facilitator of GSH biosynthesis [65]. ARD1 elevated intracellular GSH by promoting stabilization of γ-glutamylcysteine ligase catalytic subunit (*GCLC*) mRNA, thereby supporting proliferation and suppressing ferroptosis. Inhibition of *ARD1* further enhanced sorafenib-mediated ferroptosis in patient-derived xenograft (PDX) models.

In a separate genome-wide knockout screen in IFN-γ-treated HCC cell lines, cAMP response element-binding protein regulated transcription coactivator 3 (*CRTC3*) was found to protect HCC cells from ferroptosis and to weaken the antitumor activity of IFN-γ [66]. Subsequent 2D cell line validation showed that depletion of *CRTC3* remodeled lipid metabolism, increased polyunsaturated fatty acid content, and sensitized HCC cells to lipid peroxidation, ferroptosis inducers, and IFN-γ. This sorafenib-sensitizing phenotype was further supported in a subcutaneous HCC xenograft model, where *CRTC3*-knockout tumors showed stronger growth inhibition after sorafenib treatment.

#### 3.1.3. Antioxidant Buffering Programs in Therapy Resistance

Beyond glutathione metabolism, redox homeostasis can also be maintained through mitophagy-linked antioxidant programs. In this context, *LINC01607*, identified through RNA-seq of lenvatinib-resistant HCC cells combined with genome-wide CRISPR/Cas9 screening, promoted lenvatinib resistance by upregulating p62, enhancing protective mitophagy, and activating the p62–NRF2 antioxidant axis. This resistance mechanism was further supported by xenograft and PDO validation, showing that *LINC01607* silencing restored lenvatinib sensitivity [67].

Further supporting the role of redox buffering in therapy resistance, an in vivo CRISPR/Cas9 screen using the human GeCKOv2 library, which targets both protein-coding genes and miRNAs, identified *miR-3689a-3p* as a determinant of sorafenib sensitivity in HCC [68]. Enrichment of sgRNAs targeting *miR-3689a* in sorafenib-treated xenografts indicated that *miR-3689a-3p* loss promotes survival under therapeutic pressure. Mechanistically, *miR-3689a-3p* targeted copper chaperone for superoxide dismutase (CCS) and disrupted superoxide dismutase type 1 (SOD1)-dependent mitochondrial antioxidant defense. Clinically, *miR-3689a-3p* was downregulated in human HCC tissues and associated with favorable prognosis in patients with HCC, consistent with its role in modulating redox adaptation.

#### 3.1.4. Iron Metabolism and Ferroptosis-Related Therapeutic Vulnerabilities

Iron availability catalyzes lipid peroxidation through Fenton chemistry, making iron metabolism a critical determinant of ferroptosis susceptibility. A 2D genome-wide CRISPR/Cas9 screening identified ferredoxin 1-like (*Fdx1l*) as a survival dependency in HCC [69]. *FDX1L* was upregulated in tumors and associated with poor prognosis, with its functional relevance further supported by validation in HCC cell lines, patient-derived primary cells, and subcutaneous and orthotopic tumor models. Its depletion disrupted Fe-S cluster maintenance, triggered an iron-starvation response, increased iron uptake, and caused iron overload, lipid peroxidation, and ferroptosis, highlighting *FDX1L* as a promising therapeutic target for HCC.

In addition, an in vivo CRISPR screen in cell-derived HCC xenograft models identified POU class 3 homeobox 3 (*POU3F3*)-driven retinoic acid metabolism as a protective regulator of sorafenib-induced ferroptosis. This finding was further validated in HCC cell lines, xenograft models. In addition, pharmacological inhibition of POU3F3 with rosarin synergized with sorafenib in preclinical models [17].

Overall, these studies indicate that ferroptosis resistance in HCC is sustained by interconnected defenses involving GPX4 stability, glutathione metabolism, antioxidant signaling, and iron handling. Targeting these oxidative stress-buffering mechanisms may provide an effective strategy to enhance the therapeutic activity of sorafenib, lenvatinib, and immunotherapy in HCC.

### 3.2. Metabolic Adaptation and Nutrient Dependencies Sustain HCC Growth

Beyond dedicated ferroptosis-defense pathways, the ability of HCC cells to maintain redox homeostasis is tightly intertwined with broader metabolic programs. CRISPR-based functional screens have increasingly shown that nutrient utilization, bioenergetic plasticity, and anabolic adaptation collectively provide a critical foundation for tumor survival, proliferation, and therapeutic resistance in HCC.

#### 3.2.1. Glycolysis and Mitochondrial Bioenergetic Plasticity

An in vivo genome-wide CRISPR/Cas9 screen in an orthotopic HCC model identified kelch repeat and BTB domain containing 11 (*KBTBD11*) as a suppressor of metabolic adaptation in HCC [70]. Functional studies in HCC cell lines and 3D spheroid models demonstrated that KBTBD11 suppresses malignant phenotypes in vitro, while orthotopic liver tumor models confirmed its tumor-suppressive role in vivo. Mechanistically, KBTBD11 promoted ubiquitin-dependent degradation of enolase 1 (ENO1), thereby restraining glycolysis, lowering ATP and lactate levels, increasing reactive oxygen species, and enhancing apoptosis. These findings highlight a connection between the ubiquitin–proteasome system and metabolic regulation in liver cancer, providing mechanistic insight into HCC progression.

More specifically, an in vitro kinome-focused CRISPR screen further showed that loss of dual-specificity tyrosine phosphorylation-regulated kinase 1A (*DYRK1A*) enhanced sensitivity to the oxidative phosphorylation (OXPHOS) inhibitor IACS-010759 [71]. This vulnerability was validated in HCC cell lines and further supported by subcutaneous xenograft models, PDOs, and PDXs. Mechanistically, DYRK1A phosphorylated SMAD3 to blunt TGF-β signaling and maintain solute carrier family 1 member 5 (*SLC1A5*) expression, thereby supporting glutamine uptake under OXPHOS stress.

Complementing this observation, a genome-wide screen in HCC cell lines identified mitochondrial translation genes as essential for HCC growth, and this vulnerability was further supported by in vivo xenograft validation, highlighting tigecycline as a therapeutic agent to disrupt respiratory chain biogenesis [72]. In tigecycline-refractory cells, excessive EREG/AREG–EGFR–ERK1/2–MYC signaling sustained glycolysis, creating vulnerability to combined MEK or EGFR inhibition.

#### 3.2.2. Amino Acid Metabolism and Nutrient-Responsive AKT Regulation

Consistent with the importance of amino acid metabolism, a cell-based CRISPRi screen identified TAR (HIV-1) RNA-binding protein 1 (*TARBP1*) as a critical regulator of glutamine dependence in cancer cells. This hit was validated in HCC cell lines and subcutaneous xenografts, showing that TARBP1 promotes tumor growth by methylating and stabilizing selected tRNAs, thereby sustaining ASCT2 translation and glutamine uptake. Moreover, both TARBP1 and ASCT2 are co-upregulated in HCC tissues and associated with poor prognosis [74].

In addition, a cell-based CRISPR screen identified TNF receptor-associated factor 5 (*TRAF5*) as the E3 ligase mediating amino acid-induced AKT degradation, thereby mechanistically linking high-protein diets to tumor suppression and chemosensitization in liver cancer models [73]. Importantly, the physiological relevance of this mechanism was further validated in *Traf5*-deficient mice and liver cancer mouse models. Together, these findings indicate that amino acid availability not only supports anabolic metabolism, but also reshapes oncogenic signaling through nutrient-responsive regulatory circuits.

#### 3.2.3. Lipid Metabolism and Cholesterol-Driven Proliferative Signaling

Beyond amino acid metabolism, lipid-associated metabolic signaling has also been functionally defined by in vivo CRISPRi screening, which uncovered a cholesterol–TAZ–TEAD2–ANLN/KIF23 pathway that promotes tumor cell proliferation. This dependency was supported in HCC cell models and immunocompetent mouse models, in which *ANLN*/*KIF23* depletion or pharmacological inhibition of cholesterol/TEAD signaling suppressed tumor growth and improved responses to sorafenib or immune checkpoint blockade [15]. These findings suggest that lipid-associated signaling can create therapeutically actionable dependencies beyond its conventional role in membrane biogenesis and energy storage.

Collectively, these studies indicate that HCC is sustained by multilayered metabolic dependencies spanning glycolysis, mitochondrial respiration, glutamine utilization, cholesterol-driven proliferative signaling, and nutrient-responsive AKT regulation. More importantly, CRISPR-based screens reveal that these pathways do not operate in isolation, but instead form adaptive and compensatory networks that enable tumor growth under metabolic and therapeutic stress, thereby exposing actionable metabolic vulnerabilities in HCC.

### 3.3. Autophagy, Lysosomal Homeostasis, and Organelle Quality Control Support Viability and Stemness

Another highly recurrent theme is the dependence of HCC cells on autophagy–lysosomal pathways and organelle quality control systems. A cell-based CRISPR activation screen targeting membrane-associated proteins identified thioredoxin-related transmembrane protein 2 (*TMX2*) as a driver of HCC viability and tumorigenesis [75]. This dependency was validated in HCC cell lines and subcutaneous xenograft models, where TMX2 promoted macroautophagy by facilitating KPNB1 nuclear export and TFEB nuclear import, while also enhancing cytoprotective mitophagy through interaction with VDAC2 and VDAC3 to recruit PRKN to defective mitochondria during oxidative stress. Clinically, TMX2 is upregulated in HCC tissues, associated with poor prognosis, and its knockdown enhances lenvatinib efficacy.

Complementing this, a cell-based metabolic CRISPR knockout screen identified ATPase H+-transporting V1 subunit D (*ATP6V1D*) as a key regulator of HCC stemness that maintains autophagic flux through lysosomal acidification and promotion of CHMP4B–IST1 interaction and ESCRT-III assembly [76]. This hit was supported by HCC cell line assays and in vivo tumor models, in which *ATP6V1D* depletion suppressed stemness and malignant progression, whereas low-dose bafilomycin A1 showed therapeutic potential by targeting this lysosomal dependency.

In the context of targeted therapy resistance, an unbiased genome-wide CRISPR/Cas9 screen in Huh7 cells identified lysosomal protein transmembrane 5 (*LAPTM5*) as a key mediator of lenvatinib resistance through enhanced autophagic flux and autolysosome formation [77]. The association was subsequently validated in patient-derived cells, organoids, xenografts, immunocompetent mouse models, and clinical samples. Accordingly, hydroxychloroquine or *LAPTM5* depletion restored lenvatinib sensitivity, and LAPTM5 expression showed biomarker potential in clinical samples. Together, these studies position the autophagy–lysosome pathway as a central node linking stress adaptation, mitochondrial quality control, stemness maintenance, and drug resistance in HCC.

### 3.4. CRISPR Screens Uncover Mechanisms of Therapeutic Resistance in HCC

#### 3.4.1. RTK Rewiring and Compensatory Signaling in Lenvatinib Resistance

CRISPR screening has been effective in delineating how HCC escapes multikinase inhibitors through receptor tyrosine kinase (RTK) rewiring and downstream signaling adaptation. In acquired lenvatinib-resistant organoids and HCC cell lines, a kinase-focused CRISPR/Cas9 screen identified NIMA-related coiled-coil kinase 7 (*NEK7*) as a critical resistance gene, with support from both in vivo patient-derived organoid-based xenograft (PDOX) screening and in vitro screening in resistant HCC cell lines [51]. Functional validation in resistant PDOs, xenografts, and an immunocompetent HCC mouse model further showed that *NEK7* loss restored lenvatinib sensitivity. Mechanistically, NEK7 binds to EGFR and promotes its phosphorylation at serine 1070, thereby activating MAPK and PI3K/AKT signaling and sustaining resistant growth.

In a complementary study, in vitro CRISPR/Cas9 screening combined with transcriptome profiling identified platelet-derived growth factor receptor alpha (*PDGFRA*) overexpression as another determinant of lenvatinib resistance, and pharmacological inhibition with avapritinib resensitized resistant HCC models, including orthotopic mouse tumors, PDOs, and PDXs [78]. Similarly, a genome-scale CRISPR/Cas9 knockout screen in MHCC97L cells identified nuclear factor kappa B subunit 1 (*NFKB1*) and MET proto-oncogene (*MET*) as druggable mediators of lenvatinib resistance. These hits were validated by genetic silencing in vitro, and pharmacological targeting with quinazoline or cabozantinib restored lenvatinib sensitivity and synergistically suppressed HCC growth in both cell line assays and in vivo mouse models [79].

Beyond lenvatinib, CRISPR-based studies in MET-amplified MHCC97H cells further showed that phosphatase and tensin homolog (*PTEN*) deficiency sustains AKT signaling despite MET blockade. In parallel, capmatinib-treated MHCC97H xenograft models revealed adaptive resistance associated with PI3K–AKT enrichment and ERBB2/ERBB3 upregulation, supporting biomarker-guided combination strategies involving MET, AKT, and ERBB-directed therapies [80].

#### 3.4.2. Non-RTK Adaptive Programs in Lenvatinib-Resistant HCC

Additional lenvatinib-focused studies also pointed to non-RTK adaptive programs. Autophagy and RNA processing emerged as recurrent vulnerabilities, with LAPTM5 promoting autolysosome formation and splicing factor 3b subunit 4 (*SF3B4*)-related spliceosome dependency featured in lenvatinib-resistant tumors [50,77]. In PDO-based CRISPR screens, spliceosome dependency, particularly *SF3B4*, was shown to support HCC survival and progression. Pharmacological SF3B4 modulation further suppressed growth and enhanced lenvatinib efficacy in PDOXs. The same organoid platform identified ferroptosis defense as a resistance mechanism, with *GCLC* as a top-ranked hit. *GCLC* knockdown sensitized organoids to lenvatinib in PDOXs [50].

A separate cell-line-based CRISPRa screen identified *METTL8* (methyltransferase 8, tRNA N3-cytidine) as another driver of lenvatinib resistance [81]. The finding was validated in HCC cell models and extended to xenografts, which showed METTL8-mediated resistance in vivo. *METTL8* was enriched in resistant cells, inversely correlated with lenvatinib sensitivity in patient samples, and could be pharmacologically targeted by rabdosiin, supporting its potential as both a therapeutic target and a predictive biomarker.

At a broader level, zeste white 10 (*ZW10*) was implicated as a tumor-supportive and TKI-resistance-associated gene by cell-based CRISPR screening and in silico analyses. Database analyses linked high *ZW10* expression to poor prognosis, reduced TKI responsiveness, and altered immune infiltration [82]. However, this hit currently lacks validation in PDOs or in vivo HCC models, so its mechanistic and translational relevance remains less established than that of the resistance drivers above.

#### 3.4.3. CRISPR-Defined Resistance Mechanisms Beyond Lenvatinib

Resistance mechanisms extend beyond lenvatinib. In sorafenib-treated HCC mice, genome-wide knockout studies identified COP9 signalosome subunit 5 (*Cops5*) as a driver of resistance by stabilizing mitogen-activated protein kinase 2 (MK2) and activating heat shock protein family B (small) member 1 (HSPB1), thereby suppressing ferroptosis [83]. Inhibition of the COPS5/MK2 axis synergized with sorafenib in HCC cells, PDOs, and mouse models.

Beyond first-line agents, a CRISPR activation screen in Huh7 cells linked TGF-β receptor-associated binding protein 1 (*TGFBRAP1*) to regorafenib insensitivity and cancer stemness by stabilizing TGF-β receptor type 1 (TGFBR1) and amplifying a positive TGF-β feedback loop. Targeting this circuitry reduced stem-like features and improved regorafenib response [84]. This finding was further supported by subcutaneous xenograft models, PDOs, PDXs, and clinical cohort analyses.

Likewise, another in vitro genome-wide screen identified *ERBB3* as a synthetic lethal partner of regorafenib in sorafenib- or lenvatinib-resistant HCC cell models. Mechanistic assays in cell lines showed that ERBB3 activates the HIF1A–ABCB1 pathway, while xenograft models, PDX models, and clinical cohorts supported ERBB3-low status as a predictor of sequential regorafenib benefit. In ERBB3-high resistant tumors, the combination of seribantumab and regorafenib restored treatment sensitivity in preclinical in vivo models [85]. In parallel, a loss-of-function CRISPR screen in HepG2 cells identified Rho GTPase-activating protein 35 (*ARHGAP35*) as a negative regulator of regorafenib resistance [86]. Follow-up validation was mainly limited to 2D HCC cell line models, where *ARHGAP35* depletion activated RhoA-driven epithelial–mesenchymal transition (EMT) and conferred regorafenib resistance, while RhoA inhibition reversed EMT-associated resistance and restored drug sensitivity.

#### 3.4.4. Convergent Adaptive Programs and Therapeutic Implications

Collectively, these studies indicate that resistance to multikinase inhibitors in HCC is not driven by a single escape route, but instead arises through convergent programs involving RTK rewiring, compensatory PI3K/AKT and MAPK signaling, autophagy, spliceosome dependency, ferroptosis suppression, EMT, stemness-associated signaling, and adaptive drug efflux. Notably, CRISPR-based screening has also extended these insights beyond multikinase inhibitors. In 2D HepG2 and Huh7 cell models, genome-wide CRISPR/Cas9 screening identified antioxidant 1 copper chaperone (*ATOX1*) as a critical mediator of cisplatin resistance in liver cancer; pharmacological inhibition of ATOX1 restored cisplatin sensitivity through copper-dependent repression of NOTCH1/HES1 signaling [87]. This finding is supported by cell line, clinical association, biochemical, and in vivo xenograft validation, but lacks confirmation in PDO or PDX models. CRISPR-based functional interrogation therefore not only elucidates the mechanistic architecture of therapeutic resistance, but also provides a framework for biomarker-informed and rational combination strategies in HCC.

### 3.5. CRISPR Screens Identify Drivers of Stemness, Invasion, Metastasis, and Progression

#### 3.5.1. Autophagy- and Metabolism-Associated Regulators of HCC Stemness

Beyond therapy resistance, CRISPR-based screens have delineated the molecular programs underlying HCC stemness, progression, invasion, and context-specific therapeutic vulnerabilities. As noted above in Section 3.3, *ATP6V1D* supports HCC stemness and tumor progression through maintenance of autophagic flux [76].

A metabolic CRISPR/Cas9 knockout screen in HCC tumorspheres further identified scavenger receptor class B member 2 (*SCARB2*) as a driver of cancer stem cell-like properties [88]. Follow-up validation in 2D HCC cell lines, primary HCC cells, and PDOs showed that *Scarb2* loss reduced tumorsphere formation, marker-positive populations, and sorafenib resistance. Mechanistic studies showed that SCARB2 enhances MYC acetylation and transcriptional activity by disrupting HDAC3-mediated deacetylation at lysine 148. Screening of FDA-approved compounds nominated polymyxin B as a SCARB2-binding agent that disrupted the SCARB2-MYC interaction and reduced tumor burden, supporting SCARB2 as a therapeutic target.

#### 3.5.2. Epigenetic and Transcriptional Programs Driving Stemness and Metastasis

In parallel, genome-scale CRISPR knockout screening in in vitro CD24^+^CD133^+^ HCC stem-like cells identified SET domain-containing 1A (*SETD1A*) and trithorax group proteins as key epigenetic regulators of HCC stemness [89]. This hit was validated in HCC cell lines and sorted cancer stem cell (CSC) populations, where *SETD1A* loss reduced CSC marker-positive cells, spheroid formation, migration, invasion, EMT, and sorafenib resistance, and further supported by in vivo xenograft studies showing impaired tumor growth and prolonged survival after *SETD1A* knockdown. SETD1A was associated with poor prognosis, improved relapse prediction when combined with serum α-fetoprotein, and promoted oncogenic transcriptional programs through coordinated H3K4me3/H3K27me3 remodeling, enhancer activation, and cooperation with polyadenylate-binding protein cytoplasmic 1 (PABPC1).

A genome-wide CRISPR screen in MYC/TP53^R249S^-engineered primary human hepatocyte models identified RELA proto-oncogene (*RELA*) loss as a driver of transformation, stemness, and metastasis [90]. This hit was validated in vitro, where *RELA* ablation promoted proliferation, anchorage-independent growth, EMT, invasion, and stemness programs, and was further supported in *Fah*-deficient immunodeficient mouse models, where *RELA* loss accelerated in situ HCC formation and metastasis. Mechanistically, reduced RELA increased dishevelled segment polarity protein 1 (DVL1) expression, promoted β-catenin nuclear accumulation, and activated Wnt/β-catenin signaling. Notably, the RELA agonist betulinic acid suppressed tumor growth and metastasis in TP53^R249S^ xenograft models.

In vivo CRISPRa screen in an immunocompromised mouse model also identified X antigen family member 1B (*XAGE1B*), polo-like kinase 4 (*PLK4*), LIM domain only 1 (*LMO1*), and myeloid-associated differentiation marker like 2 (*MYADML2*) as functional drivers of HCC proliferation and lung metastasis. Follow-up in vitro validation in HCC cell lines showed that overexpression of these genes promoted proliferation and invasion. Clinically, elevated MYADML2 expression correlated with worse overall survival and decreased chemotherapy sensitivity [91].

#### 3.5.3. RNA Processing, Protein Synthesis, and Membrane Trafficking Programs in HCC Progression and Invasion

Complementing these findings, in vivo CRISPR screening identified DEAD-box helicase 41 (*DDX41*) as another facilitator of liver cancer tumorigenicity [92]. Follow-up in vitro mechanistic studies showed that DDX41 enhances R-loop resolution, ribosome biogenesis, and protein synthesis, while in vivo xenograft and hydrodynamic injection models confirmed its role in promoting tumor growth and liver cancer initiation. Importantly, DDX41-high tumors exhibited increased sensitivity to homoharringtonine, highlighting a potential biomarker-guided therapeutic opportunity.

At the level of membrane trafficking and invasion, in vitro sequential genome-wide CRISPR screens in a liver cancer-derived cell line identified *MGAT1* and *MGAT2*, and the Golgi protease *SPPL3* as regulators of tetraspanin surface presentation. Cell-based validation further showed that MGAT1/2-mediated biantennary N-glycosylation and SPPL3-dependent trafficking regulation sustain cell-surface tetraspanin abundance and promote cancer cell invasion, although their in vivo relevance remains to be established [93].

#### 3.5.4. Chromosomal Context and Synthetic-Lethal Vulnerabilities

Chromosomal context has likewise been functionally interrogated. CRISPR-mediated engineering of chr8p loss in isogenic HCC models demonstrated that this recurrent deletion promotes invasive behavior through combined dosage effects of metastasis-related genes [94]. Genome-wide viability screening further uncovered nudix hydrolase 17 (*NUDT17*) as a synthetic-lethal vulnerability in chr8p-deficient cells. This interaction was further validated in two independent HCC cell models, supporting the robustness of this vulnerability in cell-based models, although in vivo validation of the metastatic and therapeutic phenotypes remains limited.

Conversely, in vivo CRISPRa screening in MYC-driven mouse liver tumor models, followed by confirmation in an independent *Fah*^−/−^ liver injury model, identified chromosome 1q genes *Vps72*, *Gba1*, and *Mrpl9* as functional drivers of liver tumorigenesis [95]. These physiologic in vivo screens linked VPS72, GBA1, and MRPL9 to phospholipid metabolism, endosomal–lysosomal activity, and mitochondrial regulation, respectively.

#### 3.5.5. CRISPR-Guided Generation of Therapeutic Hypotheses

Beyond target discovery alone, CRISPR-enabled functional interrogation has also supported generation of therapeutic hypothesis. In an in vitro MYCN promoter-reporter HCC screening platform, MI202 was identified as a small-molecule suppressor of MYCN that preferentially inhibited malignant hepatoma cells while sparing normal hepatocytes [96]. A subsequent JHH7 cell-based genome-wide CRISPR knockout screen nominated acyl-CoA thioesterase 2 (*ACOT2*) as a functional target of MI202, linking lipid metabolic dependency to MYCN-driven oncogenicity. This axis is supported primarily at the cell-model level, with in vivo therapeutic validation still lacking.

Together, these studies show that CRISPR screening extends well beyond essentiality mapping to uncover the genetic, metabolic, epigenetic, and chromosomal determinants of HCC aggressiveness, while also revealing biomarker-defined and mechanistically actionable therapeutic opportunities.

### 3.6. Immune Evasion and Microenvironmental Remodeling Are Shaped by Tumor-Intrinsic Genes

Recent CRISPR studies have also expanded into the tumor immune microenvironment, revealing how tumor-intrinsic genes govern immune escape and responsiveness to immunotherapy.

#### 3.6.1. Chemokine-Driven T-Cell Recruitment and Immunotherapy Response

A genome-wide in vivo CRISPR screen in immunocompetent HCC mouse models identified adenylate cyclase 7 (*Adcy7*) as a novel immune modulator in HCC [97]. Follow-up validation in subcutaneous and orthotopic immunocompetent models, with immunodeficient controls, demonstrated that *Adcy7* limits HCC progression through a T-cell-dependent antitumor mechanism. Cell-based mechanistic studies further showed that human ADCY7 enhances antitumor immunity in HCC cells through nuclear activation of CCL5, which promotes CD8^+^ T-cell infiltration; this effect can be propagated to adjacent tumor cells via exosomal transfer. These findings nominate ADCY7 as a potential target for immunomodulatory therapy, supported by both physiologically relevant in vivo validation and mechanistic in vitro assays.

Similarly, an in vivo CRISPR/Cas9 screen in immunocompetent HCC models identified phosphatidylinositol-glycan biosynthesis class L (*Pigl*) as a regulator of response to lenvatinib plus PD-1 blockade in HCC [18]. Follow-up studies showed that *PIGL* loss promoted immune-dependent resistance in vivo rather than directly affecting tumor cell growth in vitro. Mechanistic in vitro assays further revealed that nuclear PIGL limited immune evasion by suppressing CCL2 and CCL20, whereas FGFR2-mediated Y81 phosphorylation blocked its nuclear import. Clinically, nuclear PIGL and PIGL-Y81 phosphorylation may serve as biomarkers for this combination therapy.

#### 3.6.2. T-Cell Killing, Antigen Presentation, and Necroptosis Control

In parallel, a genome-wide screen in tumor–T-cell co-culture identified baculoviral IAP repeat containing 2 (*Birc2*) as a mediator of resistance to immunotherapy by promoting NFκB-inducing kinase (NIK) degradation [98]. Mechanistically, BIRC2 suppresses non-canonical NFκB activity, reduces major histocompatibility complex class I (MHC-I) expression, and limits T-cell-mediated killing. In mouse models, BIRC2 inhibition was shown to enhance responses to anti-PD-1 therapy, but further validation in human HCC is warranted.

Beyond primary tumor immune evasion, an in vivo screen in murine HCC lung metastasis models identified serum/glucocorticoid regulated kinase 1 (*Sgk1*) as a key determinant of immune control during metastatic colonization [99]. This phenotype was supported by in vivo validation showing that *Sgk1* loss promotes metastatic outgrowth under CD8^+^ T-cell immunosurveillance, and by mechanistic assays demonstrating that *Sgk1*-deficient tumor cells attenuate CD8^+^ T-cell-induced, RIPK1-dependent necroptosis. Together, these findings suggest that tumor-intrinsic regulation of antigen presentation and necroptotic sensitivity critically influences T-cell-mediated immune surveillance in HCC.

#### 3.6.3. Neutrophil-Mediated Immunosuppression and Therapeutic Combination

Consistent with these observations, CRISPR screening across immunocompetent and immunodeficient mouse HCC models identified glycogen synthase kinase 3 alpha (*Gsk3a*) as another immunosuppressive factor [100]. Follow-up in vivo validation showed that *Gsk3a* promoted tumor growth preferentially under intact immunity, while mechanistic co-culture and immune-profiling assays demonstrated neutrophil recruitment and neutrophil extracellular trap (NET) formation via the NFκB/STAT3–LRG1 axis, leading to impaired cytotoxic T-cell function. Combining Gsk3a inhibition with PD-1 blockade produced synergistic antitumor effects in mouse HCC models.

Together, these studies show that CRISPR screening can resolve diverse tumor-intrinsic mechanisms of immune regulation in HCC—from chemokine-controlled T-cell recruitment to necroptosis sensitivity and neutrophil-driven immunosuppression. Moreover, they nominate biomarker-guided strategies to refine immunotherapy combinations.

### 3.7. Tumor Suppressor Pathways, Epigenetic Dependencies, and Subtype-Specific Vulnerabilities

A final important dimension of CRISPR screening in HCC lies in its ability to uncover tumor suppressors, epigenetic dependencies, and context-specific vulnerabilities that may be especially relevant to molecularly or etiologically defined disease subsets.

#### 3.7.1. Tumor Suppressor Networks and Oncogenic Signaling Restraint

An in vivo E3 ligase-focused CRISPR knockout screen using PLC/PRF/5 cells revealed *LTN1* as a frequently downregulated tumor suppressor, with in vitro and xenograft validation showing that *LTN1* restoration limited HCC growth by ubiquitinating insulin-like growth factor 2 mRNA-binding protein 1 (IGF2BP1), thereby attenuating c-MYC and IGF-1R signaling [101]. Another CRISPR-based in vitro kinome screen identified inhibitor of nuclear factor kappa B kinase subunit epsilon (*IKBKE*) as an upstream inhibitor of FOXA1. Mechanistic in vitro assays showed that IKBKE phosphorylates and suppresses FOXA1 transcriptional activity, while organoid, xenograft, and DEN-induced genetic mouse models demonstrated that IKBKE promotes hepatocarcinogenesis, with *Ikbke* disruption delaying liver tumor development. These findings nominate the IKBKE–FOXA1 axis as a potential therapeutic target [102].

#### 3.7.2. Chromatin and Transcriptional Dependencies in HCC Survival

Beyond tumor suppressor biology, epigenetics-focused CRISPR approaches have uncovered transcriptional and chromatin-based dependencies in HCC. Using an epigenome-focused CRISPR screen under 2D monolayer and 3D spheroid culture conditions, Dzama-Karels et al. identified the menin–MLL1 complex as a critical dependency for HCC survival [103]. A secondary CRISPR screen with the menin inhibitor SNDX-5613 revealed enhanced cell death upon nuclear transcription factor Y subunit beta (*NFYB*) knockout, indicating cooperation between menin–MLL1 and NF-Y in regulating oncogenic transcription. However, these conclusions are currently supported by 2D/3D in vitro and mechanistic chromatin-profiling data, with no in vivo validation reported in that study.

#### 3.7.3. Stress-Adaptive ER Response and Translational Control

A genome-wide in vitro CRISPR screen identified RB-binding protein 8 (*RBBP8*) as a regulator of ER stress-induced ATF4 activation, protein synthesis, and stress-associated proliferation [104]. *RBBP8* loss attenuated ATF4/CHOP and XBP1s activation in cultured cells, while hepatocyte *RBBP8* suppression protected mice against tunicamycin-induced liver injury, providing in vivo support in a liver injury model. Consistently, both RBBP8 and ATF4 were upregulated in human liver cancer tissues, particularly in proliferating Ki67-positive tumor cells, suggesting that this pathway supports tumor survival under stress, although direct in vivo validation in HCC tumor growth models remains lacking.

#### 3.7.4. Etiology-Linked Vulnerabilities in NAFLD-HCC

In parallel, an in vitro CRISPR/Cas9 screen using an Epi-drug library targeting genes encoding FDA-approved drug targets and epigenetic regulators identified tubulin beta 4B class IVb (*TUBB4B*) as a selective dependency in NAFLD-HCC [61]. TUBB4B was overexpressed in human NAFLD-HCC tissues and associated with poor prognosis, and its genetic or pharmacological inhibition induced apoptosis and senescence while suppressing tumor growth in HCC organoids and NAFLD-HCC mouse models. Notably, pharmacological inhibition of TUBB4B with the FDA-approved drug mebendazole recapitulated these effects and further synergized with the Bcl-xL inhibitor navitoclax, supporting a potential combination strategy for NAFLD-HCC.

Taken together, these studies extend the scope of CRISPR screening beyond canonical oncogenic dependency mapping, showing that it can also resolve tumor-suppressive networks, chromatin and transcriptional addictions, stress-adaptive circuitry, and etiology-linked liabilities. These findings provide a framework for more precise biomarker-guided and combination therapeutic strategies in HCC.

## 4. Discussion

CRISPR-based functional screening has emerged as a powerful approach to accelerate HCC research by enabling systematic and scalable identification of tumor dependencies. As highlighted throughout this review, recent screening studies have uncovered multilayered biological programs that drive HCC progression and therapeutic resistance, including ferroptosis defense, metabolic adaptation, autophagy–lysosomal homeostasis, stemness maintenance, immune evasion, and adaptive signaling rewiring. Collectively, these findings support a model in which HCC is sustained not simply by individual oncogenic alterations, but by dynamic and context-dependent vulnerability networks that are amenable to functional interrogation and therapeutic targeting.

A major strength of the studies reviewed here is their capacity to uncover actionable vulnerabilities that would be difficult to predict from genomic analyses alone. CRISPR-based approaches have identified resistance mechanisms involving GPX4 regulation, glutathione metabolism, antioxidant buffering, iron homeostasis, metabolic plasticity, and compensatory RTK–PI3K/AKT–MAPK signaling. Moreover, the growing adoption of specialized screening modalities—including FACS-based phenotypic screens, organoid-based platforms, in vivo screens, and combinatorial perturbation strategies—has broadened the scope of functional interrogation beyond cell-autonomous fitness to encompass drug response, microenvironmental adaptation, and immune-related phenotypes.

Despite these advances, several important limitations should be considered. Most published screens have been performed in a limited set of established HCC cell lines, which only partially capture the etiological, molecular, metabolic, and immune heterogeneity of human HCCs. Thus, current findings should be viewed as context-specific functional modules rather than a comprehensive or universal atlas.

The strengths and limitations of different experimental platforms also warrant careful consideration. Model selection is another critical issue. Although 2D cell lines remain the most scalable and reproducible platforms for genome-scale screening, they incompletely recapitulate tumor architecture, metabolic gradients, extracellular matrix interactions, and stromal or immune influences. More physiologically relevant systems, including 3D cultures, organoids, and in vivo models, can better capture selected aspects of tumor organization, patient-specific biology, and host-derived selective pressures. However, these platforms also introduce challenges related to scalability, representation of the native tumor microenvironment, species-specific differences, and immune compatibility. In particular, the lack of robust CRISPR screening platforms integrating patient-derived HCC material with an intact human immune context remains an important translational gap in the field.

Technical challenges intrinsic to pooled CRISPR screening also remain relevant. Off-target activity, variable sgRNA efficiency, copy-number-associated artifacts, incomplete editing, and clonal bottlenecks—particularly in vivo—can confound hit identification and interpretation. These issues underscore the need for adequate library coverage, biological replicates, multiple independent sgRNAs per gene, and orthogonal validation using genetic, pharmacological, transcriptomic, or epigenomic approaches. Particular caution is required when interpreting hits involving chromatin regulators, as their perturbation may produce indirect transcriptional effects, pleiotropic fitness defects, or temporally dynamic phenotypes that are not fully captured by endpoint-based pooled screens [105,106,107].

Intratumoral heterogeneity represents another major challenge, as bulk pooled screens quantify average effects and may miss dependencies restricted to rare subclones or transient cell states. This limitation is particularly pertinent to HCC, where stemness, epithelial–mesenchymal plasticity, ferroptosis susceptibility, immune evasion, and drug tolerance are often state-dependent. Although single-cell CRISPR screening has not yet been widely applied in HCC, it offers a promising strategy to link perturbations with transcriptional states and to resolve dynamic and state-specific dependencies [53,108,109,110].

Precision-editing-based screening represents another promising frontier. Whereas conventional knockout or transcriptional perturbation screens map gene-level dependencies, base and prime editing can interrogate clinically relevant point mutations, allele-specific resistance mechanisms, and functional noncoding variants without introducing DNA double-strand breaks [111,112,113,114]. Although their application in HCC remains limited, continued improvements in efficiency, fidelity, and scalability may enable functional maps of HCC at single-nucleotide resolution.

Notably, most of the current CRISPR screening studies employed gene knockout approaches, which ensures high perturbation efficiency but fails to evaluate dosage effects of gene modulation. Such caveats might be complemented by epigenome editors (e.g., CRISPRi) or RNA-targeting tools (e.g., Cas13 and RNA interference). In addition, the systematic and pioneering efforts by the Achilles Project have also provided useful benchmarks for distinguishing broadly essential genes from HCC-enriched or context-specific vulnerabilities [48,115].

## 5. Future Priorities for CRISPR Screening in HCC

Future studies should prioritize the following aspects: (i) broader representation of HBV-, HCV-, alcohol-, and NAFLD-associated HCC models to reduce etiological and molecular model bias; (ii) development of physiologically relevant and immune-compatible screening platforms, such as PDOs, organoid–immune co-cultures, ex vivo tumor slices, immunocompetent models, and humanized mouse systems, to better bridge functional discovery and clinical translation; (iii) improved control of technical artifacts, including off-target effects, variable sgRNA efficiency, copy-number-associated false positives, incomplete editing, and clonal bottlenecks; (iv) time-resolved, single-cell, and multi-omic perturbation strategies to capture dynamic and state-specific dependencies; (v) precision-editing-based screens, including base and prime editing, to enable single-nucleotide level interrogation of clinically relevant mutations and resistance alleles; and (vi) integration with clinical, genomic, epigenomic, metabolomic, spatial, and pharmacological datasets to support biomarker-guided and context-specific therapeutic prioritization.

## 6. Conclusions

In summary, CRISPR-based screening has added a functional genomics dimension to HCC research by enabling systematic discovery of causal dependencies underlying tumor progression and therapeutic resistance. Although current evidence remains constrained by model limitations and does not fully capture the heterogeneity and immune complexity of human HCC, emerging context-aware, single-cell, multi-omic, and precision-editing-based approaches are poised to refine the functional mapping of genetic drivers and vulnerabilities in HCC. Ultimately, the translational value of CRISPR screening may lie in supporting biomarker-guided and context-specific therapeutic prioritization.

## Figures and Tables

**Table 1:** Recent CRISPR screening studies in HCC-related experimental models.

Relevant Biological Process	Representative Genes	Perturbation Type	Library	Model	Condition	Phenotypic Outcome	Validation Level ^a^	Ref.
Ferroptosis Resistance and Oxidative Stress Buffering	*TRIM34*	Knockout	Genome-wide	Huh7	In vitro, erastin treatment	*TRIM34* loss increased erastin-induced ferroptosis	Nude mouse xenograft, experimental lung metastasis model	[62]
	*MALT1*	Knockout	Genome-wide	PLC/PRF/5	In vitro, FACS	Enhanced ferroptosis sensitivity upon *MALT1* inhibition, particularly in combination with sorafenib	Subcutaneous xenograft, PDOs	[63]
	*PLAG1*	Knockout	Genome-wide	Hep3B, SNU-398	In vitro, erastin or sorafenib treatment	*PLAG1* loss enhanced erastin- and sorafenib-induced ferroptosis sensitivity	Subcutaneous xenograft, orthotopic xenograft	[64]
	*ARD1*	Knockout	Genome-wide	Huh7	In vitro, erastin treatment	*ARD1* loss impaired GSH biosynthesis and increased ferroptosis sensitivity	PDXs	[65]
	*CRTC3*	Knockout	Genome-wide	HepG2, SK-Hep-1	In vitro, IFN-γ treatment	*CRTC3* loss increased sensitivity to IFN-γ-induced cell death in HCC cells	Subcutaneous xenograft	[66]
	*LINC01607*	Knockout	Genome-wide	Hep3B	In vitro, lenvatinib treatment	*LINC01607*-mediated p62 upregulation promoted mitophagy and lenvatinib resistance	Subcutaneous xenograft, PDOs	[67]
	*miR-3689a-3p*	Knockout	Genome-wide	MHCC97L	In vivo, sorafenib treatment	Loss of *miR-3689a-3p* confers sorafenib resistance	Orthotopic HCC mouse models	[68]
	*Fdx1l*	Knockout	Genome-wide	Hepa1-6	In vitro, cell fitness	*Fdx1l* depletion induces ferroptosis and suppresses HCC growth	Subcutaneous xenograft, orthotopic liver tumor model	[69]
	*POU3F3*	Knockout	Genome-wide	Hep3B, JHH7	In vivo, RSL3 or sorafenib treatment	*POU3F3*-driven RA metabolism promoted resistance to ferroptosis agonists	Subcutaneous xenograft	[17]
Metabolic Adaptation and Nutrient Dependencies	*KBTBD11*	Knockout	Genome-wide	Huh7	In vivo, cell fitness	*KBTBD11* loss promoted HCC tumor growth in vivo	3D spheroid, orthotopic liver cancer model	[70]
	*DYRK1A*	Knockout	Kinases	Huh7	In vitro, IACS-010759 treatment	*DYRK1A* loss sensitized HCC cells to OXPHOS inhibition	Subcutaneous xenograft, PDOs, PDXs	[71]
	*GFM1*, *MRPL4*, *MRPS23*	Knockout	Genome-wide	HepG2, SNU-398	In vitro, cell fitness	*GFM1*, *MRPL4*, and *MRPS23* are required for liver cancer cell proliferation and mitochondrial translation	Immunocompetent mouse model, subcutaneous xenograft	[72]
	*TRAF5*	Knockout	E3 ubiquitin ligase	293T	In vitro, FACS	*TRAF5* depletion stabilizes AKT by prolonging its half-life	Immunocompetent mouse model, subcutaneous xenograft	[73]
	*TARBP1*	CRISPRi	1918 protein-coding genes	B16-F10	In vitro, glutamine-basic medium	*TARBP1* depletion suppresses HCC growth through impaired glutamine uptake	Subcutaneous xenograft	[74]
	*Anln*, *Kif23*	CRISPRi	YAP/TAZ/TEAD target genes	Mouse	In vivo, CCl4 injection	*Anln* or *Kif23* depletion suppresses HCC formation and growth	Immunocompetent mouse model	[15]
Autophagy, Lysosomal Homeostasis, and Organelle Quality Control	*TMX2*	CRISPRa	Membrane-associated proteins	Huh7	In vitro, cell fitness	*TMX2* promotes HCC growth by sustaining autophagy and mitophagy	Subcutaneous xenograft	[75]
	*ATP6V1D*	Knockout	Metabolic-related genes	PLC/PRF/5	In vitro, FACS	*ATP6V1D* sustains HCC stemness and malignant progression	Subcutaneous xenograft, metastasis mouse model, PDOs	[76]
	*LAPTM5*	Knockout	Genome-wide	Huh7	In vitro, lenvatinib treatment	*LAPTM5* promoted lenvatinib resistance by enhancing autophagic flux	Immunocompetent mouse model, subcutaneous xenograft, PDCs, PDOs	[77]
Therapeutic Resistance	*NEK7*	Knockout	Kinases	PDO	In vivo, lenvatinib treatment	*NEK7* promoted acquired lenvatinib resistance	Immunocompetent mouse model, subcutaneous xenograft, PDOs, PDOXs	[51]
				Hep3B	In vitro, lenvatinib treatment			
	*PDGFRA*	Knockout	Genome-wide	SMMC-7721, SNU-449	In vitro, lenvatinib treatment	*PDGFRA* inhibition sensitizes HCC to lenvatinib	Orthotopic HCC mouse model, PDOs, PDXs	[78]
	*NFKB1*, *MET*	Knockout	Genome-wide	MHCC97L	In vitro, lenvatinib treatment	Loss of *NFKB1* or *MET* sensitize HCC cells to lenvatinib	Subcutaneous xenograft, orthotopic HCC mouse model	[79]
	*PTEN*, *NF1*	Knockout	Genome-wide	MHCC97H	In vitro, capmatinib treatment	Loss of *PTEN* or *NF1* drives resistance to MET inhibitors	Subcutaneous xenograft	[80]
	*SF3B4*	Knockout	Genome-wide	PDO	In vitro, cell fitness	*SF3B4* drives HCC survival and oncogenicity	PDOs, PDOXs	[50]
	*GCLC*				In vitro, lenvatinib treatment	*GCLC* loss restores lenvatinib sensitivity		
	*METTL8*	CRISPRa	Genome-wide	HepG2	In vitro, lenvatinib treatment	*METTL8* promotes lenvatinib resistance	Subcutaneous xenograft	[81]
	*ZW10*	Knockout	Genome-wide	Huh7	In vitro, anlotinib treatment	*ZW10* promotes HCC cell fitness	No in vitro or in vivo validation	[82]
	*Cops5*	Knockout	Genome-wide	H22	In vivo, sorafenib treatment	*Cops5* promotes sorafenib resistance	Subcutaneous xenograft, orthotopic HCC model, PDOs	[83]
	*TGFBRAP1*	CRISPRa	Genome-wide	Huh7	In vitro, regorafenib treatment	*TGFBRAP1* promotes HCC regorafenib resistance	Subcutaneous xenograft, PDCs, PDOs	[84]
	*ERBB3*	Knockout	Genome-wide	Huh7	In vitro, regorafenib treatment	*ERBB3* drives resistance to sequential regorafenib after sorafenib or lenvatinib resistance	Subcutaneous xenograft, PDOs, PDXs	[85]
	*ARHGAP35*	Knockout	Genome-wide	HepG2	In vitro, regorafenib treatment	*ARHGAP35* depletion promotes regorafenib resistance	Cell line only	[86]
	*ATOX1*	Knockout	Genome-wide	HepG2, Huh7	In vitro, cisplatin treatment	*ATOX1* promotes cisplatin resistance	Subcutaneous xenograft	[87]
Drivers of Stemness, Invasion, Metastasis, and Progression	*SCARB2*	Knockout	Metabolic-related genes	HCCLM3	In vitro, tumorspheres	*SCARB2* maintains stem cell-like properties	Subcutaneous xenograft, orthotopic HCC model, PDOs, PDXs	[88]
	*SETD1A*	Knockout	Genome-wide	PLC	In vitro, FACS	*SETD1A* promotes HCC stemness, tumor initiation, progression, recurrence	Subcutaneous xenograft	[89]
	*RELA*	Knockout	Genome-wide	Primary human hepatocytes	In vivo, cell fitness	*RELA* acts as a context-dependent tumor suppressor	Subcutaneous xenograft, orthotopic HCC model	[90]
	*XAGE1B*, *PLK4*, *LMO1*, *MYADML2*	CRISPRa	Genome-wide	HepG2	In vivo, cell fitness	Promotes HCC tumor growth, and lung metastasis	Cell line only	[91]
	*DDX41*	Knockout	Differentially expressed genes in HCC	Huh7	In vivo, cell fitness	*DDX41* promotes HCC tumor growth, liver cancer initiation	Subcutaneous xenograft, orthotopic liver cancer model	[92]
	*SPPL3*	Knockout	Genome-wide	MEC, JHH5	In vitro, FACS	*SPPL3* maintains cell-surface tetraspanin expression	Cell line only	[93]
	*NUDT17*	Knockout	Genome-wide	HLF	In vitro, cell fitness	Chr8pLOH-specific vulnerability in liver cancer	Cell line only	[94]
	*Zbtb7b*, *Vps72*, *Gba1*, *Mrpl9*	CRISPRa	Amplified and up-regulated genes in HCC	Mice	In vivo, tumorigenesis	Promote MYC-driven liver tumorigenesis	Orthotopic HCC model	[95]
	*ACOT2*	Knockout	Genome-wide	JHH7	In vitro, MI202 treatment	Candidate lipid-metabolism-related mediator of MI202 response	Cell line only	[96]
Immune Evasion and Microenvironmental Remodeling	*Adcy7*	Knockout	Genome-wide	Hepa1-6	In vivo, cell fitness	Tumor-suppressive, immune-potentiating factor in HCC	Subcutaneous and orthotopic immunocompetent mouse models, humanized mouse	[97]
	*Pigl*	Knockout	Metabolic-related genes	Hepa1-6	In vivo, lenvatinib and anti-PD-1 treatment	*Pigl* functions as a tumor-suppressive immune modulator in HCC, and its depletion confers resistance to lenvatinib plus anti-PD-1 therapy	Immunocompetent and T-cell-deficient mouse models, orthotopic HCC model, PDOs	[18]
	*Birc2*	Knockout	Genome-wide	Hepa1-6	In vitro, T-cells treatment	*Birc2* promotes HCC immune evasion	Subcutaneous and orthotopic immunocompetent mouse models, subcutaneous immunodeficient mouse model	[98]
	*Sgk1*	Knockout	Genome-wide	Hepa1-6	In vivo, cell fitness	*Sgk1* loss promotes HCC metastatic colonization	Immunocompetent and T-cell-depleted mouse models	[99]
	*Gsk3a*	Knockout	Disease-related immune gene	Hepa1-6	In vivo, cell fitness	*Gsk3a* promotes HCC immune evasion	Subcutaneous immunocompetent and immunodeficient mouse models, orthotopic HCC model	[100]
Tumor Suppressor Pathways, Epigenetic Dependencies, and Subtype-Specific Vulnerabilities	*LTN1*	Knockout	E3 ligase	PLC/PRF/5	In vivo, cell fitness	Tumor suppressor in HCC	Subcutaneous xenograft	[101]
	*IKBKE*	Knockout	Kinases	293T	In vitro, FACS	Oncogenic inflammatory kinase in HCC	Subcutaneous xenograft, immunocompetent mouse model, PDOs	[102]
	*MEN1*, *ASH2L*, *KMT2A*	Knockout	Chromatin-mediated genes	HLF, PLC/PRF/5	In vitro, cell fitness	Disruption of the menin–MLL1 chromatin regulatory complex impaired HCC cell survival and proliferation	2D cell line, 3D spheroid	[103]
	*RBBP8*	Knockout	Genome-wide	SEM	In vitro, FACS	*RBBP8* loss impaired cell-cycle progression and protein synthesis	Immunocompetent mouse model	[104]
	*TUBB4B*	Knockout	Drug targets and epigenetic regulators	HKCI2, HKCI10	In vitro, cell fitness	*TUBB4B* supports NAFLD-HCC/HCC cell growth and tumor progression	Subcutaneous xenograft, orthotopic HCC model, PDOs	[61]

^a^ The validation level indicates the highest level of experimental or translational support reported in each study, rather than all validation assays performed. Abbreviations: PDCs: patient-derived cells; PDOXs: patient-derived organoid-based xenograft; PDXs: patient-derived xenograft.

## Data Availability

No new data were created or analyzed in this study. Data sharing is not applicable to this article.

## Abbreviations

The following abbreviations are used in this manuscript:

HCC	Hepatocellular carcinoma
HBV	Hepatitis B virus
HCV	Hepatitis C virus
NAFLD	Nonalcoholic fatty liver disease
CRISPR	Clustered regularly interspaced short palindromic repeats
Cas9	CRISPR-associated nuclease 9
CRISPRa	CRISPR activation
CRISPRi	CRISPR interference
TKI	Tyrosine kinase inhibitor
sgRNAs	Single-guide RNAs
indels	Insertions or deletions
dCas9	Nuclease-dead Cas9
FACS	Fluorescence-activated cell sorting
2D	Two-dimensional
3D	Three-dimensional
PDO	Patient-derived organoid
PDOX	Patient-derived organoid-based xenograft
PDCs	Patient-derived cells
EMT	Epithelial–mesenchymal transition
CSC	Cancer stem cell
NET	Neutrophil extracellular trap
PD-1	Programmed cell death protein-1
GSH	Glutathione
RTK	Receptor tyrosine kinase
PDX	Patient-derived xenograft
MHC-I	Major histocompatibility complex class I
KO	knockout
scRNA-seq	single-cell RNA sequencing
crRNA	CRISPR RNA
OXPHOS	oxidative phosphorylation
